# Antibiotic Profiling of E. coli Borne UTI Infection in Tertiary Healthcare Settings

**DOI:** 10.7759/cureus.56632

**Published:** 2024-03-21

**Authors:** Raman Muthusamy, Saisri Mahesh, Christy Travasso

**Affiliations:** 1 Microbiology, Saveetha Institute of Medical and Technical Sciences, Chennai, IND; 2 Medicine, Saveetha Institute of Medical and Technical Sciences, Chennai, IND

**Keywords:** public health, medical, health care, multidrug resistance, antimicrobial susceptibility

## Abstract

Introduction

In general, with frequent recurrence of urinary tract infections (UTIs), long-term antibiotic therapy is stipulated at a low dose. With this type of situation and with easy access to several classes of antibiotics in tertiary health care settings, the use of such drugs results in the development of resistant bacteria in patients. *Escherichia coli *is a frequent cause of UTI observed. Hence, it was proposed in the present study to assess the antimicrobial resistance status of *E. coli* in UTI-infected patients.

Methods

This study was conducted among female patients diagnosed with UTI. About 80 urine samples were collected in an aseptic condition, Under the process of culture identification 44 samples were found to be positive for UTI infection. The positive samples were plated on blood agar. Out of 44 samples, 18 samples were found to be positive, and 26 samples were negative for *E. coli* infection. The 18 samples were screened on MALDI-TOF for identification. Further, the samples were assessed for susceptibility to antibiotic medication within the study area.

Result

The study identified different strains of* E. coli, *and the CHB gene *E. coli was* found in eight samples. The sample showed pink oval-round spots in the culture medium and was resistant to nitrofurantoin, cephalosporin, and cephalexin antibiotics. Hence, antimicrobial susceptibility tests are necessary for managing and treating bacterial *E. coli* infections.

Conclusion

*E. coli* is a common bacterium found in the vaginal region of patients, suggesting a potential infection. *E. coli* can be associated with UTIs in women. The results from this study conclude that *E. coli* is rapidly becoming multidrug-resistant, as only higher antibiotics can inhibit its growth. To effectively manage infections caused by* E. coli* proper diagnosis, laboratory testing, and antibiotic treatment are required.

## Introduction

The Enterobacteriaceae family includes the bacterium *Escherichia coli*. The main ailments associated with pathogenic strains of *E. coli *in urinary tract infections (UTIs). Some UTIs have strains of *E. coli *that can cause UTIs [1], typically by entering the urethra and ascending into the urinary tract. Frequent urination, a burning feeling when urinating, and unclear or bloody urine are all typical signs of UTIs [2]. Infections caused by *E. coli* in patients can lead to UTIs, pelvic inflammatory disease (PID), and vaginal infections [3]. UTIs can cause symptoms such as frequent urination, painful urination, and cloudy or bloody urine. PID infects the reproductive organs and causes pelvic pain, fever, abnormal vaginal discharge, and infertility if left untreated.

*E. coli* strains that produce specific toxins, such as Shiga toxin-producing *E. coli* (STEC), can cause gastrointestinal infections. These infections can lead to symptoms such as diarrhea which may be bloody, abdominal pain, and vomiting. In severe cases, complications like hemolytic uremic syndrome (HUS) can occur. Consumption of contaminated food or water can lead to foodborne illnesses caused by certain strains of *E. coli*. The symptoms can vary but often include diarrhea, abdominal cramps, and nausea. In rare cases, *E. coli* can cause meningitis in newborns. This occurs when the bacteria enter the bloodstream and reach the central nervous system, leading to inflammation of the protective membranes surrounding the brain and spinal cord. It is a serious condition requiring immediate medical attention [4].

The majority of *E. coli *strains are safe and are a typical part of the gut flora, despite the fact that some strains of the bacteria can cause harm. They play a beneficial role in digestion and nutrient absorption. Preventing *E. coli*-related illnesses involves practicing good hygiene, such as thorough handwashing, properly cooking and handling food, and ensuring safe water sources. Prompt medical attention and appropriate treatment are necessary for individuals experiencing symptoms associated with *E. coli* infections [5].

Antibiotic resistance has become one major healthcare problem, UTIs have been associated with high-rate treatment failure, *E. coli *and is a gram-negative bacterium that can produce a large number of beta-lactam enzymes, making them resistant to most beta-lactam antibiotics [6]. The choice of antibiotic depends on factors such as the site and severity of infection, antibiotic susceptibility testing, and patient-specific factors (e.g., allergies, pregnancy). Commonly prescribed antibiotics for *E. coli* infections include fluoroquinolones, cephalosporins, and trimethoprim/sulfamethoxazole. In cases of severe *E. coli* infections, such as those causing gastrointestinal symptoms or systemic involvement, it is important to manage fluid and electrolyte imbalances [7]. This may involve oral or intravenous fluid administration, as well as monitoring and correcting electrolyte levels. Depending on the specific symptoms and complications associated with the* E. coli *infection, additional supportive care measures may be employed. Women are commonly diagnosed with *E. coli *infections using a combination of clinical examination, medical history analysis, and laboratory investigations [8-11]. Timely and accurate diagnosis is crucial for initiating appropriate treatment to manage *E. coli* infections effectively. The purpose of this work is to study the bacterial* E. coli* flora in patients.

## Materials and methods

Sample collection

The study was done at Saveetha Medical College and Hospital, Chennai, Tamil Nadu, after obtaining ethical committee clearance (356/03/2023/UG/SRB/SMCH) (IRB No.112101140). About 80 urine samples from a middle-aged group (35-50 years old) of female patients in Tiruvallur district at Saveetha Medical College and Hospital were taken into account to screen for *E. coli *from the samples. Female patients between the age of 35-50 years old, with or without symptoms of UTI, and no history of antibiotic treatment exceeding two weeks before the day of specimen collection were included. Women presenting with diabetes symptoms and having taken antibiotics within the past one or two weeks before sample collection were excluded from the study. About 5 mL of urine samples were collected in a container and the samples were transferred within two hours to the microbiology laboratory for culture and sensitivity test at Saveetha Medical College and Hospital.

Culture and identification

According to standard laboratory procedure [12], out of 80 urine samples collected from outpatients and inpatients with symptoms suggestive of UTIs. A total of 44 samples were found to be positive for UTI, loopful of urine samples were streaked on blood agar plates (Himedia, India) and incubated for 24 hours at 37 °C. After incubation, the *E. coli*. colonies typically appear smooth, small, non-hemolytic, and pink on blood agar. In that, 18 samples were positive for *E. coli,* and negative plates were further incubated for an additional 24 hours in which 26 samples were found to be still negative for *E. coli* and they were discarded. From the Blood agar plate, the pink-colored bacterial colonies were isolated and transferred into an Eppendorf tube containing a viral transport medium. The 18 *E. coli*-positive sample tubes were stored in an ice-gel container to be transported to Gujarat Biotechnology and Scientific Research Centre, Gujarat Gandhinagar, for MALDI-TOF screening. The bacterial isolates were inoculated onto a MALDI-TOF target plate, wherein the matrix solution (α-cyano-4-hydroxycinnamic acid) was applied and then allowed to air dry. Target plates were examined using a MALDI-TOF mass spectrometer to identify the bacteria. The bacterial pathogens are identified using the concept of mass-to-charge ratios (m/z) present in bacterial proteins by MALDI-TOF screening.

Antimicrobial susceptibility

An antimicrobial susceptibility test was performed by the Kirby Bauer [13] disc diffusion method to determine the resistance (R%) [14]. The following drug discs were tested: nitrofurantoin (5 µg), cephalosporin (5 µg), and cephalexin (5 µg). The above antibiotics were chosen in concordance with the prescription pattern followed at the study site. Then a loopful of bacterial isolates from the above-cultured colony was taken and transferred into a tube containing nutrient broth, the loopful of the bacterial isolate culture was swabbed onto Muller Hinton agar (MHA) using a sterile cotton swab. The antibiotic disc was placed on the agar plate and incubated for 24 hours at 37 °C. The antimicrobial activity was then determined by measuring zones of inhibition, the inhibition zones appeared to be clear on the agar plate and the collected data was analyzed by Microsoft Office Excel.

## Results

Antibiotic susceptibility test

**Figure 1:**
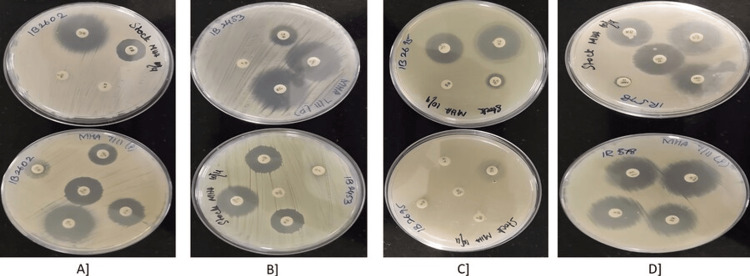
Antibiotic susceptibility testing by disc diffusion method (A) 1B2602, (B) 1B2453, (C) 1B2695, (D) 1R578

The agar disc diffusion method was used to assess antibiotic resistance in *E. coli* strains isolated from UTI cases. Specifically, four positive strains (1B2602, 1B2453, 1B2695, 1R578) were selected, as depicted in Figures 1A-1D. Among these strains, 1B2602 demonstrated sensitivity to linezolid and cefdinir but was resistant to ciprofloxacin and penicillin. Strain 1B2453 exhibited sensitivity to gentamycin and linezolid, while being resistant to cotrimoxazole and penicillin. Strain 1B2695 showed sensitivity to cefoxitin and ciprofloxacin but was resistant to cefdinir, cotrimoxazole, erythromycin, and penicillin. Strain 1R578 was sensitive to cefepime and linezolid but resistant to ampicillin. Overall, the result showed that selected *E. coli* strains were found to be sensitive to linezolid and resistant to penicillin as summarized in Table 1.

**Table 1:** Antibiotic susceptibility test and zone of inhibition * Sensitive, -- Resistant

Sl.No.	Antibiotics		Zone of Inhibition (mm)			
			Sample Code			
			1B2602	1B2453	1B2695	1R578
1.	Ampicillin (AMP)		-	-	-	7
2.	Amikacin (AK)		-	-	-	20
3.	Cefepime (CPM)		-	-	-	23^*^
4.	Cefdinir (CD)		24^*^	16	--	-
5.	Cotrimoxazole (COT)		18	--	--	21
6.	Cefoxitin (CX)		12	13	21^*^	18
7.	Ceftriaxone (CTR)		-	-	-	20
8.	Ciprofloxacin (CIP)		--	19	20^*^	-
9.	Erythromycin (E)		17	7	--	-
10.	Gentamicin (GEN)		13	27^*^	10	-
11.	Linezolid (LZ)		25^*^	26^*^	11	22^*^
12.	Meropenem (MRP)		-	-	-	19
13.	Penicillin (P)		--	--	--	-
14.	Vancomycin (VA)		14	15	-	-

MALDI-TOF screening

About 44 urine samples from female patients were reported as positive for UTI 10µL of the sample was cultured on blood agar plates, and 44 plates were sent for analysis using molecular techniques, particularly MALDI-TOF, at the Gujarat Biotechnology and Scientific Research Centre, Gujarat Gandhinagar. Out of 44 plates, 26 plates were negative for *E. coli* infection and 18 were taken into consideration for the study. The various strains of E. coli found in the urine samples are depicted in Table 2. The *E. coli* strain that was most frequently discovered was the CHB gene *E. coli*. In the group of chitinolytic enzymes, chitobiase (CHB) is necessary for the generation of N-acetyl-D-glucosamine from the chitin biopolymer. Inclusion bodies were formed in the majority of overexpressed CHB in the *E. coli *expression system.

**Table 2:** Different strains of E. coli found in the urine samples

Sl. No.	Sample strains	Oraganism best match
1	E. coli ATCC 25922 CHB	Closely related to Shigella/ Escherichia fergusonii
2	E. coli ATCC 25922 THL	Closely related to Shigella/ Escherichia fergusonii
3	E. coli ATCC 35218 CHB	Closely related to Shigella/ Escherichia fergusonii
4	E. coli DH5 alpha BRL	Closely related to Shigella/ Escherichia fergusonii
5	E. coli ESBL EA RSS 1528T CHB	Closely related to Shigella/ Escherichia fergusonii
6	E. coli MB11464 1 CHB	Closely related to Shigella/ Escherichia fergusonii
7	E. coli RV412 A1 201006a LBK	Closely related to Shigella/ Escherichia fergusonii
8	E. coli W3350 MMG	Closely related to Shigella/ Escherichia fergusonii
9	ESBL EA RSS 1528T CHB	Closely related to Shigella/ Escherichia fergusonii
10	ATCC 25922 THL	Closely related to Shigella/ Escherichia fergusonii
11	W3350 MMG	Closely related to Shigella/ Escherichia fergusonii
12	E. coli W3350 MMG	Closely related to Shigella/ Escherichia fergusonii
13	ATCC 25922 CHB	Closely related to Shigella/ Escherichia fergusonii
14	DSM 682 DSM	Closely related to Shigella/ Escherichia fergusonii
15	BM 114641 CHB	Closely related to Shigella/ Escherichia fergusonii
16	E. coli ATCC 25922 THL	Closely related to Shigella/ Escherichia fergusonii
17	ATCC 35218 CHB	Closely related to Shigella/ Escherichia fergusonii
18	DH5 alpha BRL	Closely related to Shigella/ Escherichia fergusonii

## Discussion

*E. coli *bacteria are known to reside in the intestines but can sometimes cause infections in female patients when they enter the urinary tract or other parts of the reproductive system. High rates of nitrofurantoin, cephalosporin, and cephalexin antibiotic resistance were found in this study conducted at Saveetha Dental College. Ciprofloxacin, gentamicin, norfloxacin, and nitrofurantoin are considered to be acceptable medications for treating *E. coli* empirically in the study field. Regular antibiotic susceptibility testing is indicated in both hospital and community settings. In summary, *E. coli* infections in patients can produce a range of symptoms and, if left untreated, may have negative effects. The antimicrobial therapy of *E. coli* infections in females is an essential aspect of managing these infections effectively. *E. coli* is a common cause of UTIs in women, and appropriate treatment is crucial to treat the infection and prevent complications.

When selecting antibiotics for *E. coli* infections, several factors should be considered. In antibiotic susceptibility, the choice of antibiotics depends on the susceptibility of the *E. coli *strain causing the infection. Local antibiogram data and susceptibility testing help guide the selection of appropriate antibiotics. It is important to choose antibiotics to which the* E. coli* strain is sensitive to ensure effective treatment. The choice of antimicrobial therapy may vary based on the site and severity of the *E. coli *infection. For lower UTIs (cystitis), oral antibiotics are commonly prescribed, while upper UTIs (pyelonephritis) may require intravenous antibiotics initially, followed by oral antibiotics for completion of therapy. Individual patient factors, such as pregnancy, allergies, and comorbidities, need to be considered when selecting antimicrobial therapy. Some antibiotics may be contraindicated during pregnancy, and alternative options may be required for patients with specific allergies or compromised renal function.

Commonly prescribed antibiotics for *E. coli *infections are fluoroquinolones (e.g., ciprofloxacin, levofloxacin), beta-lactam antibiotics (e.g., amoxicillin-clavulanate, ceftriaxone), trimethoprim/sulfamethoxazole, and nitrofurantoin. It is important to adhere to recommended treatment guidelines and complete the full course of antibiotics to ensure the eradication of the *E. coli* infection and prevent the development of antibiotic resistance.

However, it is worth noting that antibiotic resistance is a growing concern globally, including among *E. coli *strains. Resistance patterns vary geographically, and local resistance data should be considered when prescribing antimicrobial therapy. Monitoring of antibiotic resistance, judicious use of antibiotics, and adherence to antimicrobial stewardship practices are crucial in combating the development and spread of resistance. In cases of recurrent or complicated *E. coli* infections, a thorough evaluation by a healthcare professional is necessary to identify any underlying predisposing factors that may contribute to the recurrent infections. This may include imaging studies, urologic evaluations, or referral to a specialist [6]. In conclusion, appropriate antimicrobial therapy is vital in the management of *E. coli* infections in females, particularly UTIs. The selection of antibiotics should be based on susceptibility testing, site and severity of infection, and patient-specific factors. Adherence to recommended treatment guidelines and vigilance in combating antibiotic resistance is essential for effective treatment and the prevention of recurrent infections [11]. It was resistant in 91% of isolates and discovered resistance to last-resort medications such as tigecycline, colistin, and carbapenems. Among the isolates that were multidrug resistant [12]. There has only been sporadic systematic AMR monitoring of these bacteria, particularly in China's developed regions 91% of isolates had multidrug resistance, and tigecycline, colistin, and carbapenems were among the last lines of defense. they also detected a heterogeneous group of O-serogroups among the multidrug-resistant isolates from these studies we can come to the conclusion that *E. coli* is becoming a multidrug-resistant bacteria, so antimicrobial susceptibility must be improved for a better prognosis.

The study was limited by a small sample size, with patients exclusively selected from the narrow age range of 35-50 years old. Detailed histories of infection and clinical characteristics were unavailable for the selected patients. Additionally, clarity regarding the history of medication undergone was lacking. An enhanced patient record system would be advantageous for future research endeavors.

## Conclusions

The antimicrobial susceptibility of *E. coli* is crucial for effective treatment. Different strains have varying antibiotic susceptibilities, requiring careful selection of antibiotics. *E. coli *and CHB genes *E. coli* were found more in the samples. However, the highest rate of resistance by *E. coli *was found to be on the antibiotic nitrofurantoin, cephalosporin, and cephalexin. Common antibiotics include fluoroquinolones, beta-lactams, trimethoprim/sulfamethoxazole, and nitrofurantoin. The results from this study conclude that *E. coli* is rapidly becoming multidrug-resistant, as only higher antibiotics can inhibit its growth. Hence, monitoring local resistance patterns, promoting judicious use, and following treatment guidelines are essential. Continuous surveillance and the development of new strategies are necessary to combat *E. coli* antimicrobial resistance.
